# Recent common human coronavirus infection protects against severe acute respiratory syndrome coronavirus 2 (SARS-CoV-2) infection: A Veterans Affairs cohort study

**DOI:** 10.1073/pnas.2213783119

**Published:** 2022-11-07

**Authors:** Nathanael R. Fillmore, Raphael E. Szalat, Jennifer La, Westyn Branch-Elliman, Paul A. Monach, Vinh Nguyen, Mehmet K. Samur, Mary T. Brophy, Nhan V. Do, Nikhil C. Munshi

**Affiliations:** ^a^Cooperative Studies Program, Veterans Affairs Boston Healthcare System, Boston, MA 02130;; ^b^Department of Medicine, Harvard Medical School, Boston, MA 02115;; ^c^Department of Medical Oncology, Dana-Farber Cancer Institute, Boston, MA 02115;; ^d^Section of Hematology & Medical Oncology, Boston University School of Medicine, Boston, MA 02118;; ^e^Center for Healthcare Organization and Implementation Research, Veterans Affairs Boston Healthcare System, Boston, MA 02130;; ^f^Department of Biostatistics, Harvard School of Public Health, Boston, MA 02115;; ^g^Section of General Internal Medicine, Boston University School of Medicine, Boston, MA 02118;; ^h^Section of Hematology/Oncology, Veterans Affairs Boston Healthcare System, Boston, MA 02130

Solomon et al. (1) identified an association between exposure to young children and lower risk of severe Coronavirus disease 2019 (COVID-19) in adults. The authors theorize that this protection may be due to higher rates of coronavirus immunity conferred by recent exposure to and subsequent infection with other human coronaviruses (HCoVs). Cross-protection is also supported by laboratory studies (2–4).

Although laboratory and epidemiologic studies are suggestive, the clinical impact of HCoV infection on severe acute respiratory syndrome coronavirus 2 (SARS-CoV-2) susceptibility is not fully elucidated. Thus, the aim of this study was to measure the association between recent HCoV infection with common strains (HCoV-229E, HCoV-OC43, HCoV-NL63, HCoV-HKU1) and future risk of SARS-CoV-2 prior to widespread vaccination and immunity.

## Methods

We used the national Veterans Affairs (VA) electronic health record to define a retrospective cohort of patients with one or more SARS-CoV-2 microbiologic test results (polymerase chain reaction [PCR] or antigen) and one or more HCoV microbiologic test results (PCR) during the period from 20 February 2020 to 2 February 2021, the first year of the pandemic and prior to widespread SARS-CoV-2 vaccination. This study was approved by the VA Boston Research and Development Committee with a waiver for informed consent. Only patient time prior to SARS-CoV-2 infection was included. Incidence rate ratios (IRRs) of SARS-CoV-2 infection after HCoV infection were calculated, and statistical significance was evaluated using Wald tests. IRR relative to PCR-diagnosed infection with noncoronavirus respiratory pathogens (e.g., respiratory syncytial virus, rhinoenterovirus, parainfluenza) was also calculated as a negative control. To adjust for potential confounding factors, multivariable Cox proportional hazards models were fit to further assess the association between HCoV and SARS-CoV-2 infection.

## Results

**Fig. 1.**
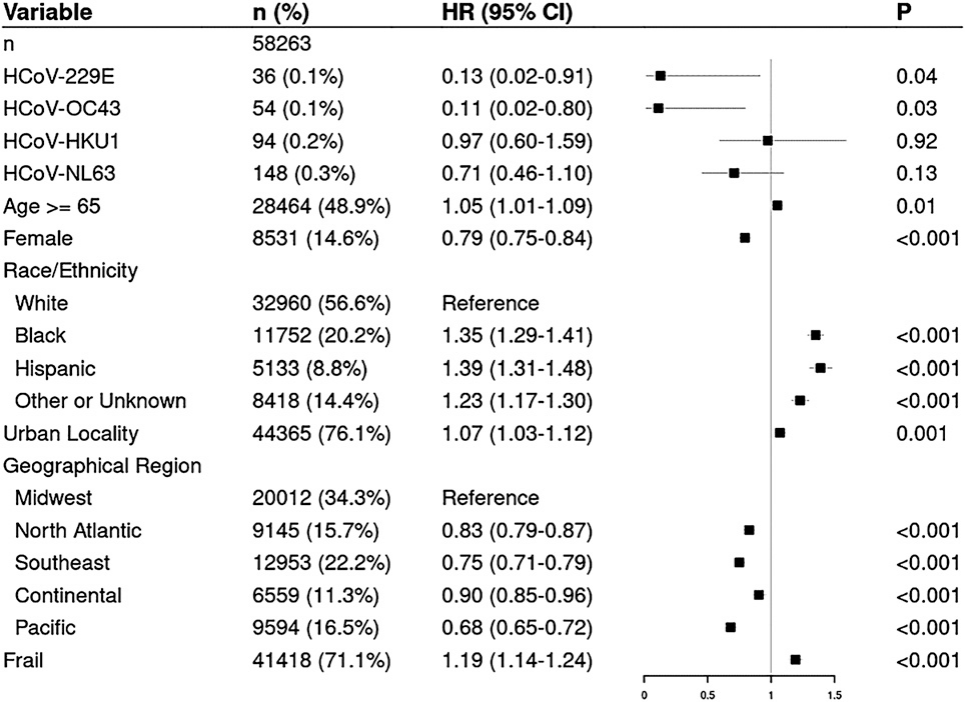
Forest plot of a multivariable Cox model of COVID-19 infection in patients recently tested for HCoV infection. The column labeled “*n* (%)” shows the number of patients in each category and the percentage of total patients in the analytic sample in each category. The column labeled “HR (95% CI)” and the corresponding plot show the hazard ratios (HRs) and 95% CIs in the multivariable Cox model. The column labeled “P” shows the *P* value as to whether the HR differs from one. Microbiologically confirmed infection with HCoV strains is coded as a time-dependent covariate; all other variables are measured at baseline. Other variable definitions are as in our prior work (7).

We identified 58,263 patients with SARS-CoV-2 and HCoV test results (Table 1). Among patients without documented HCoV infection, the overall incidence rate (IR) for SARS-CoV-2 was 267/1,000 person-years. The IR for SARS-CoV-2 was significantly lower after a positive test for 229E (37/1,000 person-years; IRR = 0.14, 95% confidence interval [CI] = 0.00 to 0.77; *P* = 0.02) or OC43 (28/1,000 person-years; IRR = 0.11, 95% CI = 0.00 to 0.59; *P* = 0.006). No risk reduction was found after infection with HKU1 or NL63 (275 and 216/1,000 person-years, respectively). SARS-CoV-2 IR after a positive test for one of the negative-control pathogens was 188/1,000 person-years (IRR not statistically different from one). Findings were similar in the multivariable analysis (Fig. 1). Following infection with 229E, the first patient was infected with SARS-CoV-2 127 d later; for OC43, the earliest SARS-CoV-2 infection was 322 d later. Distant HCoV infection during the 5-y period prior to the pandemic did not confer protection.

**Table 1.** Patient characteristics in the full analytic sample and in patients who tested positive for each HCoV strain

	Analytic sample	HCoV-229E	HCoV-HKU1	HCoV-NL63	HCoV-OC43
*n*	58,263	36	94	148	54
Age, y, median [IQR]	64.00 [50.00, 73.00]	59.50 [44.50, 72.00]	65.00 [54.25, 72.75]	61.50 [40.50, 71.00]	70.00 [56.25, 74.75]
Female (%)	8,531 (14.6)	4 (11.1)	10 (10.6)	24 (16.2)	7 (13.0)
Race/ethnicity (%)					
Non-Hispanic White	32,960 (56.6)	23 (63.9)	57 (60.6)	83 (56.1)	38 (70.4)
Non-Hispanic Black	11,752 (20.2)	4 (11.1)	16 (17.0)	27 (18.2)	7 (13.0)
Hispanic	5,133 (8.8)	4 (11.1)	10 (10.6)	18 (12.2)	3 (5.6)
Other or unknown	8,418 (14.4)	5 (13.9)	11 (11.7)	20 (13.5)	6 (11.1)
Urban	44,365 (76.1)	30 (83.3)	80 (85.1)	123 (83.1)	45 (83.3)
Region (%)					
Midwest	20,012 (34.3)	13 (36.1)	17 (18.1)	42 (28.4)	8 (14.8)
North Atlantic	9,145 (15.7)	6 (16.7)	32 (34.0)	52 (35.1)	13 (24.1)
Southeast	12,953 (22.2)	5 (13.9)	15 (16.0)	20 (13.5)	10 (18.5)
Continental	6,559 (11.3)	2 (5.6)	9 (9.6)	15 (10.1)	3 (5.6)
Pacific	9,594 (16.5)	10 (27.8)	21 (22.3)	19 (12.8)	20 (37.0)
Frail (%)	41,418 (71.1)	26 (72.2)	48 (51.1)	99 (66.9)	27 (50.0)

Variable definitions are as in our prior work (7). IQR, interquartile range.

## Discussion

In this national study, prior infection with non-SARS HCoVs provided short-term cross-protection against SARS-CoV-2; distant infections were not associated with reduced risk, consistent with prior data suggesting that immunity to HCoVs is partial and short lived (5).

These clinical data complement the findings in Solomon et al. (1), which found that adults with more frequent exposure to children were less likely to develop severe COVID-19. The authors theorize that this association may be attributed to ongoing intermittent exposure to and infection with other coronaviruses. Our study augments this theory by demonstrating a reduced risk of future SARS-CoV-2 among patients with confirmed HCoV infections. Our clinical data also align with in vitro data about cross-reactive immune responses observed with 229E and OC43 demonstrated by epitope mapping and IgG antibody titers against SARS-CoV-2 spike glycoprotein in SARS-CoV-2–uninfected individuals (6).
